# Inhibition of ACAT as a Therapeutic Target for Alzheimer’s Disease Is Independent of ApoE4 Lipidation

**DOI:** 10.1007/s13311-023-01375-3

**Published:** 2023-05-08

**Authors:** Ana C. Valencia-Olvera, Deebika Balu, Naomi Faulk, Aspasia Amiridis, Yueting Wang, Christine Pham, Eva Avila-Munoz, Jason M. York, Gregory R. J. Thatcher, Mary Jo LaDu

**Affiliations:** 1 Department of Anatomy and Cell Biology, University of Illinois at Chicago, Chicago, IL 60612 USA; 2 University of Wisconsin – Madison, Madison, WI 53706 USA; 3 Department of Pharmaceutical Sciences, University of Illinois at Chicago, Chicago, IL 60612 USA; 4 Present Address: AbbVie Inc., 1 N. Waukegan Road, North Chicago, IL 60064 USA; 5 Syneos Health, Av. Gustavo Baz 309, La Loma, Tlalnepantla de Baz, 54060 Mexico; 6 Department of Pharmacology & Toxicology, University of Arizona, 1703 E Mabel St., Tucson, AZ 85721 USA

**Keywords:** Alzheimer’s disease, *APOE4*, ACAT-1, Oligomeric Aβ, Neuroinflammation, APP processing

## Abstract

*APOE4*, encoding apolipoprotein E4 (apoE4), is the greatest genetic risk factor for Alzheimer’s disease (AD), compared to the common *APOE3*. While the mechanism(s) underlying *APOE4*-induced AD risk remains unclear, increasing the lipidation of apoE4 is an important therapeutic target as apoE4-lipoproteins are poorly lipidated compared to apoE3-lipoproteins. ACAT (acyl-CoA: cholesterol-acyltransferase) catalyzes the formation of intracellular cholesteryl-ester droplets, reducing the intracellular free cholesterol (FC) pool. Thus, inhibiting ACAT increases the FC pool and facilitates lipid secretion to extracellular apoE-containing lipoproteins. Previous studies using commercial ACAT inhibitors, including avasimibe (AVAS), as well as *ACAT*-knock out (KO) mice, exhibit reduced AD-like pathology and amyloid precursor protein (APP) processing in familial AD (FAD)-transgenic (Tg) mice. However, the effects of AVAS with human apoE4 remain unknown. In vitro, AVAS induced apoE efflux at concentrations of AVAS measured in the brains of treated mice. AVAS treatment of male E4FAD-Tg mice (5xFAD^+/-^*APOE4*^+/+^) at 6–8 months had no effect on plasma cholesterol levels or distribution, the original mechanism for AVAS treatment of CVD. In the CNS, AVAS reduced intracellular lipid droplets, indirectly demonstrating target engagement. Surrogate efficacy was demonstrated by an increase in Morris water maze measures of memory and postsynaptic protein levels. Amyloid-beta peptide (Aβ) solubility/deposition and neuroinflammation were reduced, critical components of *APOE4*-modulated pathology. However, there was no increase in apoE4 levels or apoE4 lipidation, while amyloidogenic and non-amyloidogenic processing of APP were significantly reduced. This suggests that the AVAS-induced reduction in Aβ via reduced APP processing was sufficient to reduce AD pathology, as apoE4-lipoproteins remained poorly lipidated.

**Supplementary Information:**

The online version contains supplementary material available at 10.1007/s13311-023-01375-3.

## Introduction

Lipid dysregulation is involved in the pathogenesis and progression of neurodegenerative diseases including Alzheimer’s disease (AD) [1–3]. Accumulation of neutral lipids, particularly cholesteryl esters (CE) and triacylglycerol (TAG) stored as lipids droplets (LD), is found in aged and AD brains [4–8]. In addition, brains of AD patients [9] and familial AD-transgenic (FAD-Tg) mice [4, 5] have elevated cholesterol levels. Thus, repurposing drugs targeting lipid pathways may provide effective AD therapeutics.

The greatest genetic risk factor for sporadic AD is inheritance of the ε4 allele of the human (h)-*APOE* gene compared to the common ε3 and the neuroprotective but rare ε2 allele [10]. Apolipoprotein E (apoE), the protein encoded by the *APOE* gene, is the only lipoprotein-competent apolipoprotein expressed in the brain, where it is secreted primarily by astrocytes [11, 12]. As the protein component of CNS-lipoproteins, apoE-containing lipoproteins enable lipid transport within the interstitial space of the brain, available for uptake by both neurons and glia as apoE is a ligand for receptor-mediated endocytosis by the low-density lipoprotein receptor (LDLR) family. Although expressed at comparable levels, apoE4 levels in brain are lower than apoE3 levels in both humans and Tg mouse models [13–15], suggesting that apoE4 is unstable and undergoes accelerated degradation, likely due to the observed reduced lipidation state of apoE4-lipoproteins compared to apoE3-lipoproteins [16–23].

One of the pathological hallmarks of Alzheimer’s disease (AD) is amyloid plaques composed primarily of amyloid-beta (Aβ) peptide, although Aβ also exists in soluble forms, specifically soluble oligomeric Aβ (oAβ) considered a proximal neurotoxin [24–31]. Importantly, *APOE4* is associated with accelerated Aβ accumulation, both as deposited amyloid and soluble Aβ [32–34]. Aβ peptides are produced primarily by neurons via the sequential cleavage of amyloid precursor protein (APP) by β- and γ-secretases (amyloidogenic pathway) at lipid rafts in the plasma membrane (PM) [35–37]. Outside of lipid rafts, APP is processed at the PM by α-secretase and -secretases producing sAPP-α (non-amyloidogenic pathway) [38–40]. The APP processing pathway is highly regulated by the mobilization of cholesterol between the lipid rafts and PM [41, 42]. Recent data suggest that the amount of cholesterol in neuronal lipid rafts is regulated by astrocytic apoE-lipoproteins promoting or limiting APP processing by β- and γ-secretases, and hence the amount of Aβ production [40]. ApoE is also known to interact with both amyloid plaques and soluble Aβ [43], an association that may be key in Aβ clearance [44], and studies have demonstrated that apoE4 does not clear plaques or soluble Aβ as well as apoE3 [45, 46]. Thus, increasing the lipidation of apoE4 represents a promising therapeutic target for modulating Aβ levels in the CNS.

To correct the structure of apoE4, and thus its function, several therapeutics targeted at enzymatic activities known to alter lipoprotein biogenesis and remodeling in the periphery have been explored for repurposing as CNS therapeutics [47]. Over a decade of evidence demonstrates that the cell-surface lipid transporter ATP binding cassette subfamily A member 1 (ABCA1) modulates AD pathogenesis, with ABCA1 antagonism increasing, and agonism decreasing, pathology [48–51]. We previously demonstrated that RXR agonists induce an increase in ABCA1 expression with an increase in apoE4 lipidation and a decrease in both total Aβ42 and soluble Aβ in EFAD (5xFAD^+/-^/*APOE4*^+/+^)-Tg mice [13]. However, severe hepatomegaly is associated with RXR agonism due to an induction of hepatic lipogenic pathways by sterol-regulatory element-binding protein 1C (SREBP1C) [52, 53].

Inhibition of ACAT (acyl-CoA: cholesterol-acyltransferase or sterol O-acyltransferase) is another potential pathway for increasing apoE lipidation and modifying APP processing. ACAT exists as two isoforms, ACAT1 is distributed ubiquitously, and ACAT2 is expressed primarily by enterocytes and hepatocytes [54, 55]. The endoplasmic reticulum (ER)-resident ACAT catalyzes intracellular cholesterol esterification, critical in this context as it reduces the ER-free cholesterol (ER-FC) pool by the formation of CE stored as LD [56]. By repurposing a cardiovascular drug that inhibits ACAT, specifically avasimibe (AVAS), LD formation is inhibited, allowing FC build-up, thus promoting ABCA1-mediated cholesterol efflux to nascent apoE4-lipoproteins [57–63]. ACAT activity and LD levels increase with age [64–66], AD, and *APOE4* [67, 68], suggesting low levels of FC that will limit apoE lipidation and its downstream effects. Importantly, previous research demonstrates that ACAT inhibition reduces Aβ-induced neurotoxicity in vitro and AD pathology in FAD-Tg mouse models, including significant reductions in plaque burden and gliosis (reviewed in [69, 70]). In vitro, blocking ACAT promotes formation of the autophagosome/lysosome that contributes to microglia-associated Aβ clearance ([71]; reviewed in [72]). In vivo, attenuating ACAT activity increases the levels of 24-hydroxycholesterol and decreases the content of APP and Aβ in the brain of FAD-Tg mice [73, 74]. Using non-isoform specific ACAT inhibitors like AVAS, APP processing is reduced in brains of FAD-Tg mice ([75, 76]; reviewed in [77]). A key factor influencing the effect of ACAT inhibition is its location in the ER [78], next to key players: (1) in microglia, inhibition of ACAT causes an increase in ER-FC, promoting formation of autophagosomes/lysosomes [71]; (2) in neurons, an increase in ER-FC promotes cholesterol turnover by activating ER-resident cytochrome P450 family 46 subfamily A member 1 (CYP46A1) [73] and 3) stimulates APP anchoring in the ER, thus reducing neuronal APP processing at the PM [41].

The observations reported have been confined to FAD-Tg models expressing endogenous mouse apoE (m-apoE), not addressing the role of h-apoE4 in these processes. Mouse and human apoE differ at ~ 100/300 amino acids, and CNS m-apoE lipoproteins remain uncharacterized as to size and level of lipidation. Additionally, no effects have been reported for m-apoE lipidation state with the inhibition of ACAT. Importantly, m-apoE and h-APP combine in FAD-Tg mice to produce severe Aβ pathology, pathology that is reduced by knocking-out (KO) the m-*APOE*, further reduced by knocking-in (KI) h-*APOE4*, and again reduced with h-*APOE3*-KI (review [79–87]).

This study was designed to determine the effect of ACAT inhibition by AVAS on h-apoE4 lipidation and AD pathology in male E4FAD-Tg mice [79, 88]. Our hypothesis is that in vivo, AVAS, via inhibition of ACAT, will increase the local ER-FC pool, promoting ABCA1-mediated efflux of cholesterol to nascent apoE4-lipoproteins, in addition to reducing APP processing. This correction of apoE4 structure via lipidation will restore its function in the CNS and reduce measures of AD pathology.

AVAS treatment significantly increased MWM measures of memory and postsynaptic protein levels, indicating surrogate efficacy, reduced intracellular LDs, demonstrating indirect target engagement, and reduced pathological changes in Aβ solubility/deposition, and neuroinflammation, all critical components of *APOE4*-modulated AD pathology. However, there was no increase in apoE4 levels or lipidation, while APP processing was significantly reduced. This suggests that the AVAS-induced reduction in processing of APP to Aβ was sufficient to establish surrogate efficacy and reduce AD pathology, as apoE4-lipoproteins remained poorly lipidated.

## Methods

All reagents used in the methods are listed in Table S1.

### Cell Culture

Primary glial cultures (~ 95% astrocytes, 5% microglia) were isolated from cerebral cortex (CX) of 2-day(d) old E4FAD-non carrier (5xFAD^−/−^/*APOE4*^+/+^) pups (previously described [89, 90]). Briefly, after harvest, cells reached confluency at 10–12 d in vitro (DIV) and were trypsinized and plated into two 175 cm^2^ tissue culture flasks. After 20 DIV, secondary mixed glia cultures were seeded in 96-well plates for 24 h (h) with 10% fetal bovine serum. The cultures were changed to serum-free media for 24 h prior to treatment for 6 h and 24 h with increasing concentrations of AVAS. Cell viability and secreted apoE levels were measured as described below.

### MTT for Cell Viability

Briefly, after treatment with 0.05–10.0 μg/ml AVAS for 6 h and 24 h, the media was removed, and the cells incubated with 5% MTT and processed as per the manufacturer’s instructions. The absorbance values are expressed as % of vehicle control (VC)-treated cells (% control).

### ApoE ELISA

After treatment with 0.05–10 μg/ml AVAS for 6 h and 24 h, the media was removed and apoE levels measured with an in-house apoE ELISA that utilizes α-apoE as capture antibody and α-apoE biotin-gt as the detection antibody (previously described [91]). A recombinant human apoE3 standard was prepared and diluted to produce the standard curves. Samples were diluted to read within the linear range of the standard curve.

### PK Analysis

3-month (M) old male C57BL/6 (BL6) mice (Charles River, Wilmington, MA) were treated by gavage with AVAS (30 mg/kg) for 3 h, 6 h, and 24 h, followed by sacrifice and analysis of AVAS levels in blood and brain. Briefly, stock solutions of AVAS that were dissolved in DMSO were prepared daily. The drug was formulated in 0.5% w/v sodium carboxymethyl cellulose, 9% Tween 80 in water with 1% final DMSO concentration (vehicle solution). Mice were sacrificed 3 h, 6 h, and 24 h after treatment. Spot checks to determine whether male E4FAD mice differed from male BL6 were made at 3 h with 100 mg/kg AVAS treatment by gavage, and the level of the drug was measured in both blood and brain. Mice were sacrificed using CO_2_ asphyxiation, blood was immediately drawn from the inferior vena cava, and mice were intracardially perfused with ice-cold PBS, decapitated, and brains were removed, separated into 2 hemispheres (hemis) and flash frozen. Blood samples were immediately transferred into heparin-coated tubes, centrifuged for 6 min (m) at 3220 g at 4 °C. Plasma supernatants were flash-frozen and immediately stored at − 80 °C until analysis. Brain and plasma concentrations of AVAS were quantified by LC–MS/MS analysis on a SCIEX 5500 system equipped with an Agilent 1200 HPLC, using warfarin as an internal standard [52].

### Animals, Study Design, and Treatment

All experiments follow the Institutional Animal Care and Use Committee protocols of the University of Illinois at Chicago. As previously described, the EFAD carriers are 5xFAD^+/−^/*APOE*^+/+^ and the non-carrier is 5xFAD^−/−^/*APOE*^+/+^ [13]. Male E4FAD mice (*n* = 20) were randomized into 2 treatment groups (*n* = 10): vehicle control (VC) and AVAS (AVAS). AVAS (30 mg/kg weight) was suspended at 4.8 mg/ml in vehicle solution and administered via daily gavage for 60 d from 6 to 8 M; VC-treated mice were treated with vehicle solution. Mice were weighed daily prior to gavage treatment to determine drug dose.

### Behavior and Mouse Sacrifice

In the week prior to sacrifice, mouse behavior was evaluated using the open field test (OFT) and an adapted Morris water maze (MWM) protocol (previously described [92, 93]). Behavior was tracked in real time by an overhead camera and videos analyzed using ANY-maze video tracking software (Stoelting Co., Wood Dale, IL, USA). Briefly, for OFT, the mice were placed in the center of a white box (l38.5 w × 30 h × 30 cm) and allowed to move around freely for 10 m. The total distance traveled and the time spent in the periphery and center were measured. For MWM, acquisition trials (learning) consisted of recording the latency to reach a visible platform in 4 trials/day (1 min each) for 5 consecutive days. On day 6, in the absence of the platform, reference memory was assessed with a single probe trial test recording the latency to reach the target location or target quadrant. A 24 h interval between the last training trial and the probe trial allows reference memory to be tested, independent of the short-term memory of the last training session [94]. After the probe trial, mice were sacrificed and brains were removed and dissected at the midline to produce two hemi-brains, one each for immunohistochemical and biochemicalanalysis (previously described [13]).

### Immunostaining

Serial sagittal sections (35 μm thick and separated by 280 μm) from E4FAD mouse brains were used for staining/immunostaining measures. For lipid staining, the sections were washed in TBS, mounted on glass microscope slides, and dried for 1 h. In the dark, 1X LipidSpot-610, “a fluorogenic neutral lipid stain that rapidly accumulates in lipid droplets where it becomes brightly fluorescent” (Biotium product sheet), was applied directly to the slides and incubated for 1 h (previously described [95]). The stained sections were imaged at 63X with a Zeiss Fluorescent Microscope, one field for subiculum (SB) and three fields for CX: visual CX, somatosensory CX, and frontal CX. Images were analyzed by counting the number of LD per nuclei (10–14 nuclei per field) and for the CX, averaged per mouse. For amyloid deposition, the sections were washed in TBS and stained with Thio-S. For Aβ deposition, astrogliosis, and microgliosis, sections were immunostained using MOAB-2, GFAP, and Iba1 antibodies, respectively (previously described [13, 96]). Whole sections were imaged at 10X magnification with a Zeiss Fluorescence microscope and analyzed for area covered by Thio-S, Aβ, GFAP, and Iba-1 in CX using ImageJ software.

### Plasma Lipoprotein Profiles

Blood was collected in heparin-coated tubes and centrifuged (3500 rpm, 15 m) to separate plasma. The total-, HDL-, and LDL-cholesterol and apoA1 and apoB levels in the plasma were measured using Beckman Coulter AU480 chemistry analyzer [97, 98]. ApoE was measured by in-house ELISA as previously described.

### Sequential Protein Extraction Fractions

For biochemical analysis, hemi-brains were dissected into CX and hippocampus (HP). Cortices were processed using a three-step-sequential protein fractionation method, resulting in soluble (Tris-buffered saline: TBS), non-ionic detergent (TBS + 1%Triton X-100: TBSX), and insoluble (neutralized formic acid: FA) (previously described [99]). Total protein in the TBS and insoluble extracts was quantified using the Bradford assay, and total protein in the TBSX extracts was quantified with the BCA Protein Assay (previously described [99]). Specific fractions were used for subsequent biochemical analyses.

### Western Blots

For Western blot analysis, 15 μg of protein from TBS-X fractions was separated on 4–12% Bis–Tris NuPAGE precast gels and transferred to 0.2 μm PVDF membranes. Membranes were incubated in 5% non-fat dry milk in TBS + Tween-20 (0.0625%) for 1 h and incubated overnight with primary antibodies against PSD95, drebrin, β-actin, β-tubulin, ABCA1, APP, and C-terminal fragments (CTF) at 4 °C. After 3 washes with TBS + Tween-20, membranes were incubated with HRP-conjugated secondary antibodies for 45 min, washed with TBS + Tween-20, developed with Pierce chemiluminescence reagents, and visualized with an Odyssey FC Imaging System (previously described [92, 99]).

### Native Gels

For native gels, 15 μg of protein from TBS fractions was separated on 4–20% Tris–glycine gels following the manufacturer’s instructions and transferred to 0.2 μm PVDF at 30 V for 16 h. After transfer, blots were treated with Ponceau S Staining Solution (0.1% (w/v) Ponceau S in 5% (v/v) acetic acid) to visualize the molecular mass markers. Membranes were incubated in 5% non-fat dry milk in TBS for 1 h and incubated overnight with primary goat anti-apoE antibody in 1% non-fat dry milk overnight at 4 °C, followed by HRP-conjugated secondary antibodies for 45 min in 1% non-fat dry milk, developed with Pierce chemiluminescence reagents, and visualized with an Odyssey FC Imaging System (previously described [22]).

### Aβ and IL-1β ELISAs

ELISAs for apoE (previously described) and Aβ42 levels were measured in TBS, TBSX, and FA extraction fractions, and oAβ (in-house, previously described [34]) and IL-1β in the TBS extract. oAβ was prepared and diluted to produce a standard curve (previously described [91, 100]). IL-1β was measured following the manufacturer’s instructions (previously described [96]). All samples were diluted to read within the sensitivity of the ELISAs.

### Statistical Analysis

All data are expressed as mean ± SEM. Data were analyzed by Student’s *t*-test using GraphPad Prism version 8 (for Mac, GraphPad Software, La Jolla, CA). With significance defined as *p* < 0.05, † is the treatment effect from comparison of the VC- to AVAS-treatment.

## Results

### AVAS Induces a Dose-Dependent Increase in Secreted apoE Levels in Primary Glial Cultures

**Fig. 1:**
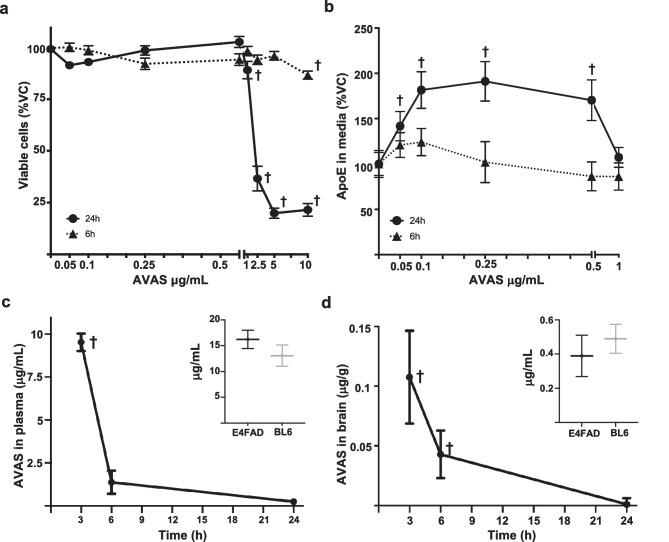
In vitro screening and pharmacokinetics of AVAS. In vitro*:* Mixed glial cultures from *EFAD-non-carrier (5xFAD*^*−/−*^*/APOE*^+*/*+^*)* mice treated with vehicle control (VC) or AVAS (0.05–10 μg/mL) for 6 h or 24 h. **a** Cell viability after AVAS treatment measured by MTT. **b** ApoE levels in the media after AVAS treatment measured by apoE ELISA. In vivo: **c** Plasma concentration of AVAS (30 mg/kg) by gavage in male BL6 mice after 3 h, 6 h, and 24 h treatment. Insert **c** Plasma concentration of AVAS (100 mg/kg) by gavage in male E4FAD (black) or male BL6 (gray) mice after 3 h treatment. **d** Brain concentration of AVAS (30 mg/kg) by gavage in male BL6 mice after 3 h, 6 h, and 24 h treatment. Insert **d** Brain concentrations of AVAS (100 mg/kg) by gavage in male E4FAD (black) or male BL6 (gray) mice after 3 h treatment. AVAS was quantified by LC–MS/MS analysis. Data are expressed as mean ± SEM (*n* = 4–8), analyzed by Student’s *t* test: *p* < 0.05, ✝ = vs. VC

To determine the toxicity of AVAS in vitro, primary glial cells were treated for 6 h and 24 h with 0.05–10.0 μg/ml AVAS (Fig. 1a). For both the 6 h and 24 h incubations, an AVAS dose from 0.05–1.0 μg/ml was not toxic. From 2.5–10.0 μg/ml, there was a small but significant reduction in viability for the 6 h treatment while viability for 24 h treatment dropped to ~ 25%. Primary glial cells treated for 24 h with non-cytotoxic concentrations of AVAS (0.05–1.0 μg/ml) induced a 50% (0.05 μg/ml) and 2-fold (0.1 and 0.5 μg/ml) increase in apoE levels in the media, with no treatment-effect at 6 h (Fig. 1b). These results demonstrate that ACAT inhibition by AVAS can promote an increase in extracellular apoE levels in glial cells and/or promote apoE stability in the media of glial cells.

### AVAS Reaches Effective Concentrations in Mouse Brain

AVAS has previously been administered to FAD-Tg mice by subcutaneous pellet [76]. To establish an effective oral dose for in vivo experiments, pharmacokinetics (PK) in plasma and brain were correlated with the AVAS-induced increase in apoE in glial cell cultures, an in vitro pharmacodynamic (PD) measure significant at 100 nM AVAS (Fig. 1b). Administration of AVAS by gavage (30 mg/kg p.o.) yielded plasma concentrations of 9.0 μg/ml at 3 h and 1.25 μg/ml at 6 h post-administration (Fig. 1c). Although the brain/blood bioavailability ratio was low (≈0.01), measured brain concentrations were above the desired PD threshold at 3 h and 6 h (Fig. 1d). To our knowledge, this is the first measurement of AVAS concentrations in the brain after oral drug delivery, showing that total brain concentrations reach 100 ng/g at 3 h after administration. To control for potential difference between the wildtype mice (male C57BL/6) used for PK and the male E4FAD mice to be used in the study, 100 mg/kg AVAS was administrated, and brain and plasma concentrations measured, with no significant differences at 3 h in plasma (inset Fig. 1c) or brain (inset Fig. 1d).

### AVAS Treatment Has Minimal Peripheral Effects on Plasma Lipid Profiles and Mouse Body Weights

**Fig. 2:**
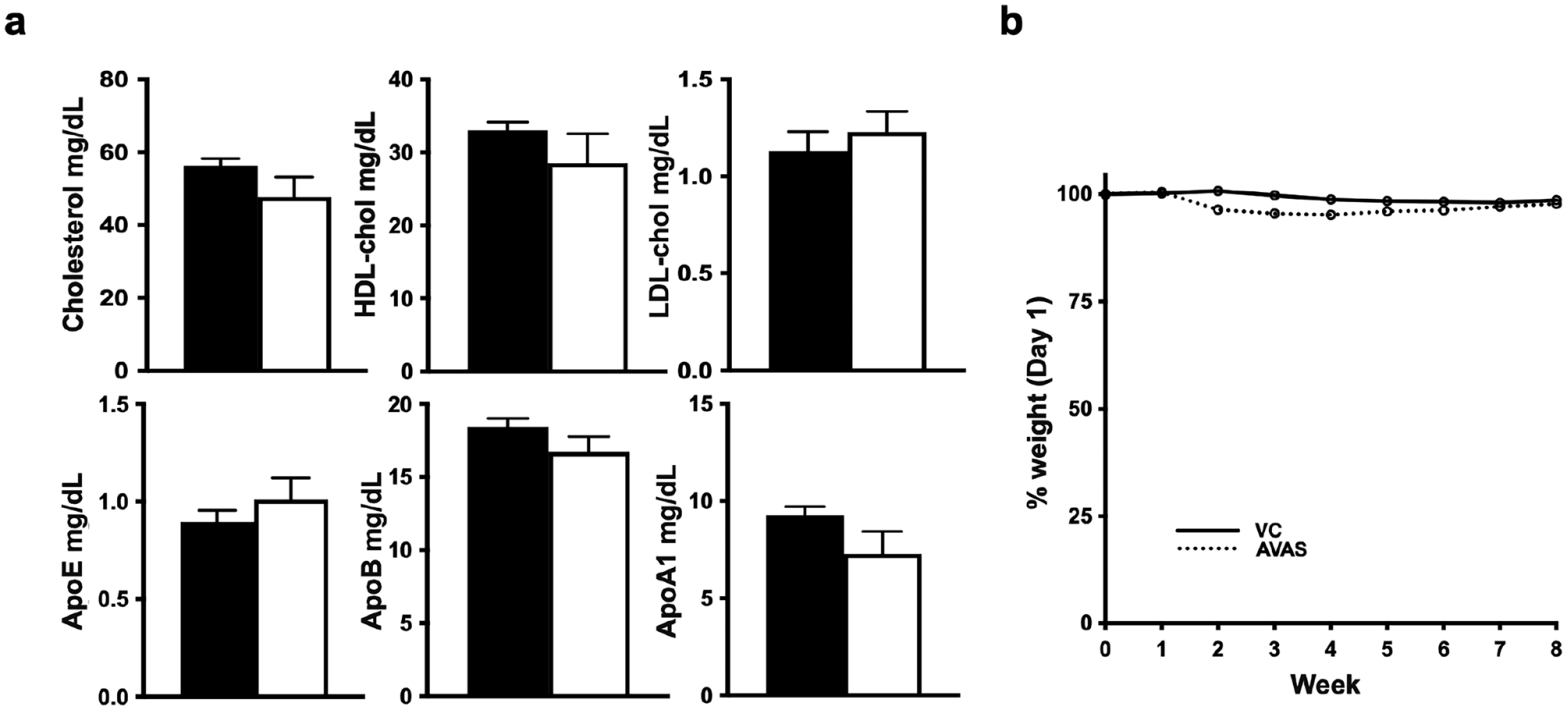
Peripheral effects of AVAS: No change in plasma lipoprotein profile or mouse body weights with AVAS treatment. **a** Plasma levels of total-, HDL-, and LDL-cholesterol measured by enzymatic assay (top) and apoE, apoB, and apoA1 measured by turbidimetry (bottom). Data are expressed as mean ± SEM (*n* = 10), analyzed by Student’s *t* test, *p* < 0.05, ✝ = vs. VC. **b**. Mouse weight expressed as % weight on day 1 of treatment. Body weight expressed as mean ± SEM (*n* = 10), analyzed by 2-way ANOVA

AVAS is a drug repurposed from the cardiovascular field. It was initially designed to promote reverse cholesterol transport, reducing atherosclerotic plaques via a reduction in blood cholesterol levels [101]. AVAS treatment is expected to increase the levels of HDL while reducing LDL, thus reducing total plasma cholesterol [102]. AVAS treatment did not modify the cholesterol-specific plasma lipid profile, including total-, HDL-, and LDL-cholesterol levels (Fig. 2a, top). As well, there was no change in peripheral levels of apoE, apoB, or apoA1 (Fig. 2a, bottom). In the AVAS-treated mice compared to VC, body weights decreased significantly but to a maximum of 5% from week 2–6, with no significant difference in weeks 7–8 (Fig. 2b). These data suggest that the AVAS-specific effects observed in this study are confined to the CNS, independent of potential peripheral effects.

### MWM Memory Performance Improved, and Postsynaptic Protein Levels Increased with AVAS Treatment

**Fig. 3:**
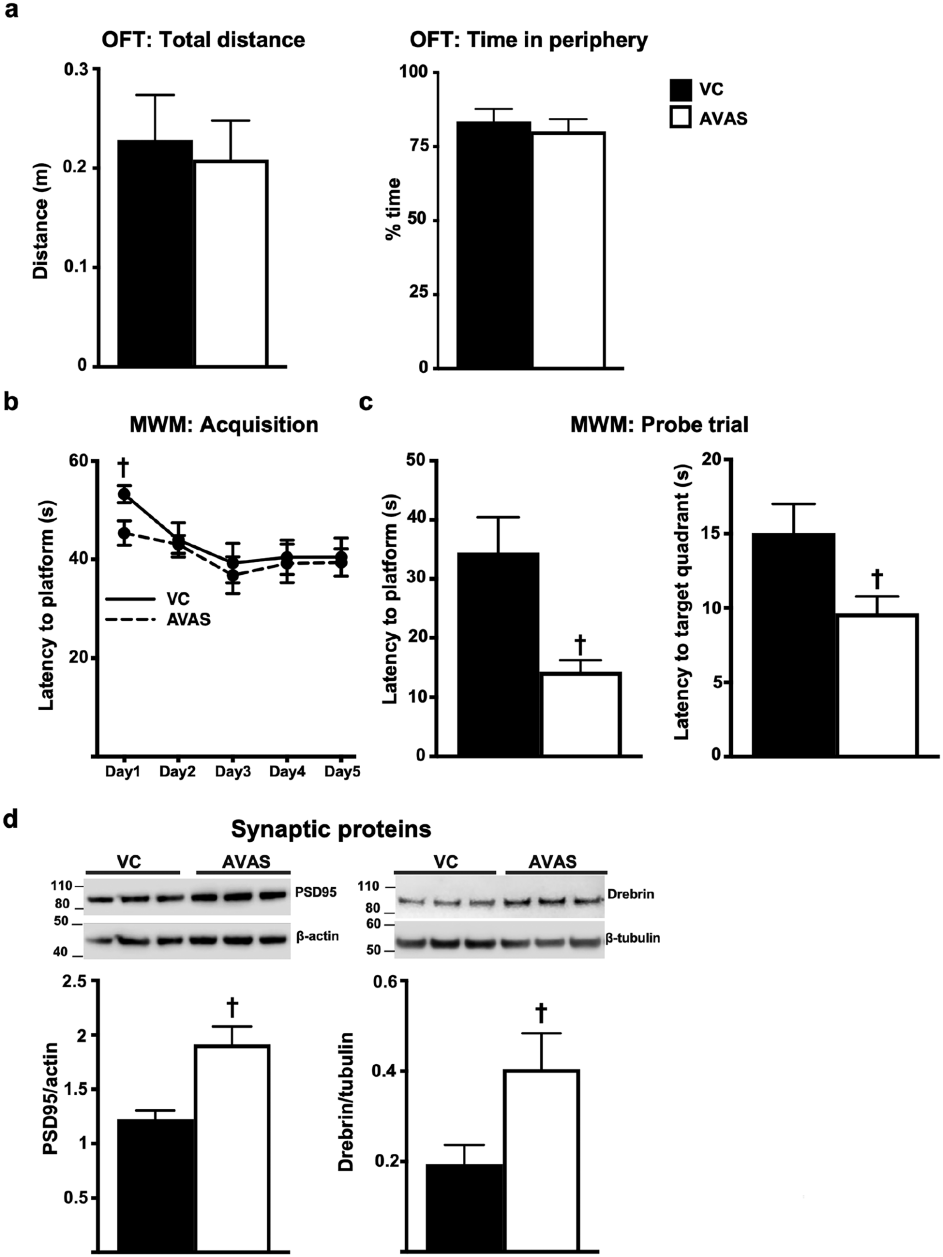
MWM measures of memory improve, and postsynaptic protein levels increase with AVAS treatment. **a** Open field (OFT) measure of total distance covered and % time in the periphery. **b** MWM acquisition/learning latency by training day **c** probe/memory trial latencies to target platform (left) and to target quadrant (right). **d** Western blot (top) for synaptic protein levels in the CX normalized to β-actin for quantification of PSD95 (left) and drebrin (right). Data are expressed as mean ± SEM (n = 10), analyzed by Student’s *t* test, *p* < 0.05, ✝= vs. VC

For an AD therapeutic, efficacy is defined as enhanced or retained cognition. We operationally defined surrogate efficacy via MWM performance + synaptic protein levels. Based on results from OFT, AVAS did not affect the general locomotion of the mice as the total distance traveled and the time spent in the periphery by AVAS-treated mice did not significantly differ from the VC mice (Fig. 3a). With the MWM, all the mice learned to reach the platform with no significant differences between the VC- and AVAS-treated mice during the acquisition trials (Fig. 3b). However, in the probe trials (memory), AVAS induced a significant ~ 50% decrease in the latency to target platform and to target quadrant compared to VC group (Fig. 3c). Representative Western blot of synaptic proteins, our second defined component of efficacy, are quantified and normalized to β-actin and β-tubulin (Fig. 3d). Levels of the post-synaptic proteins PSD95 and drebrin are significantly increased 50% and 2-fold, respectively, with AVAS treatment compared to VC. Thus, the AVAS-induced improvement in MWM memory is consistent with the increase in post-synaptic proteins, indications that AVAS exhibits surrogate efficacy.

### Intracellular Lipid Droplets Are Reduced by AVAS Treatment

**Fig. 4:**
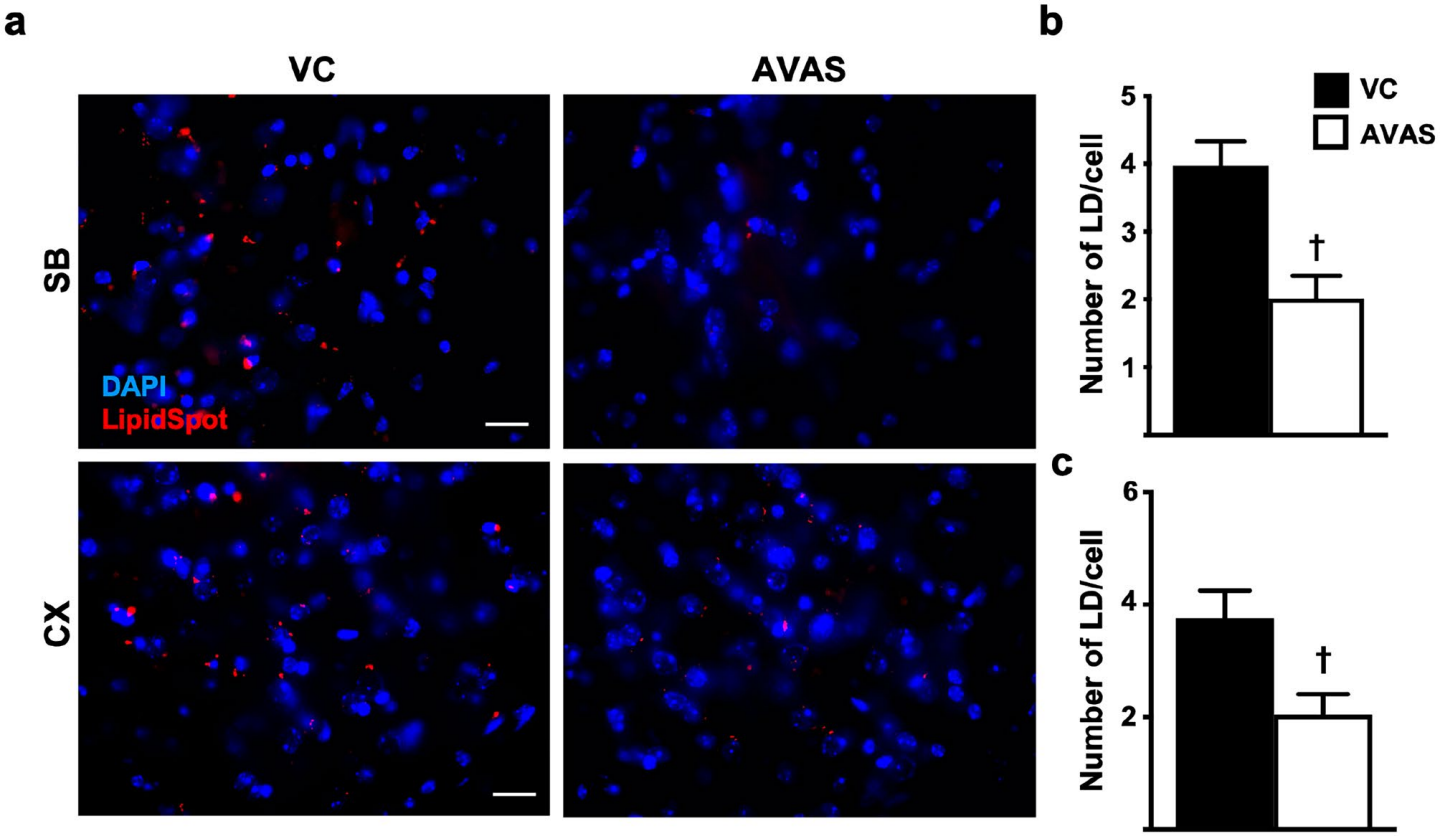
Intracellular lipid droplets are reduced by AVAS treatment. **a** Representative images of staining for lipid droplets (LD) (LipidSpot) and nuclei (DAPI) in subiculum (SB, top) and cortex (CX, bottom) for VC (left) and AVAS (right) treatment. Scale bars: 50 mm. **b** Quantification of LD per nuclei in SB (**b**) and CX (**c**). Data are expressed as mean ± SEM (*n* = 3), analyzed by Student’s *t* test: *p* < 0.05, ✝ = vs. VC

As ACAT catalyzes intracellular cholesterol esterification, facilitating the formation of intracellular LD [103], inhibition of AVAS is predicted to reduce intracellular LD. Thus, to determine target engagement for AVAS, the number of LD/cell was measured in the subiculum (SB) and CX. Figure 4a shows representative 63X sagittal images from SB (left) and CX (right) from VC (top) and AVAS (bottom) of treated mice stained with LipidSpot (LD) and DAPI (DNA). Compared to VC, AVAS induced a significant ~ 50% decrease in LD/cell in both the SB (Fig. 4b) and CX (Fig. 4c), an indication that AVAS exhibits direct target engagement.

### Deposited, Soluble, and Insoluble Aβ Are Reduced by AVAS Treatment

**Fig. 5:**
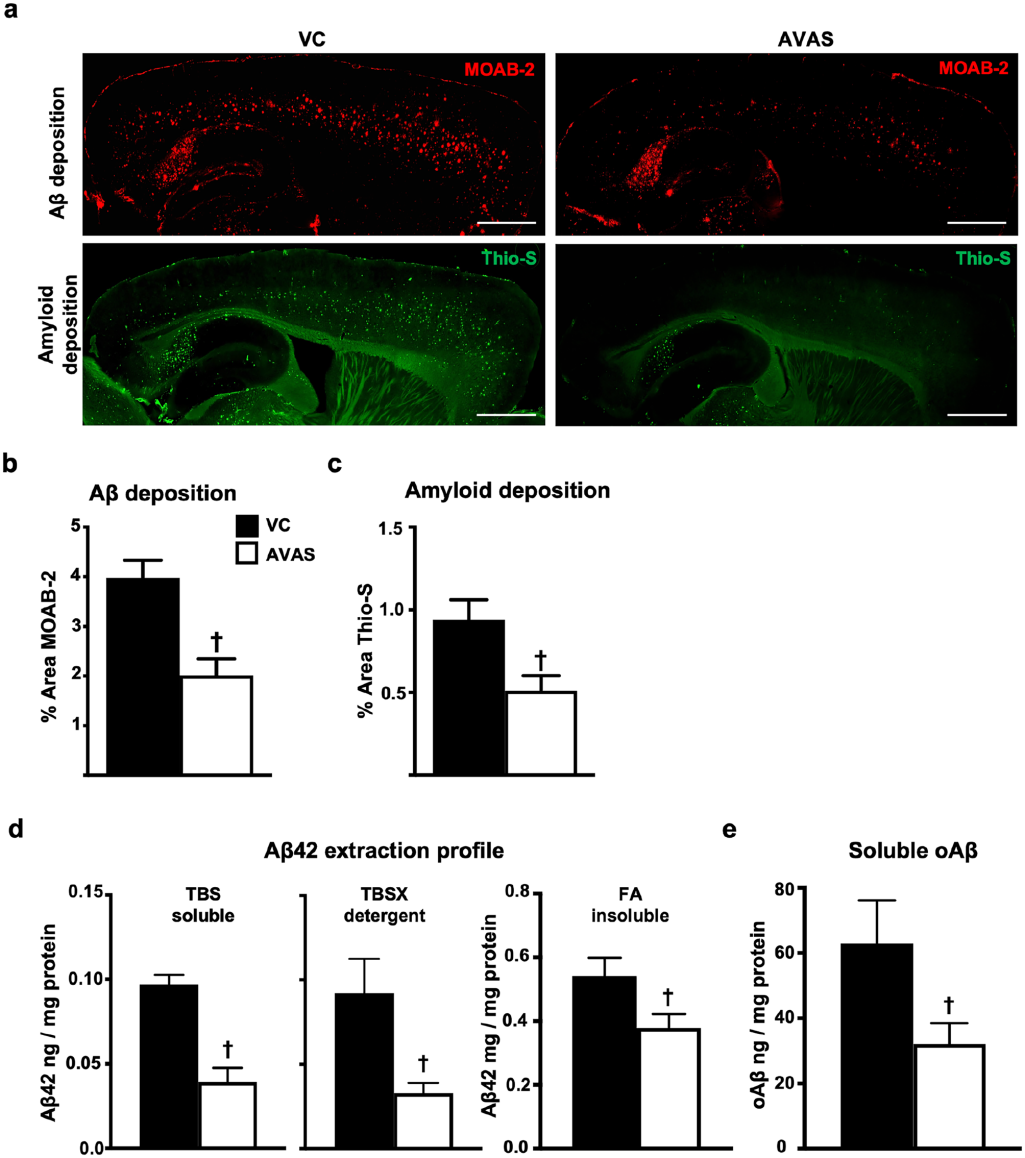
Deposited, soluble, and insoluble Aβ are reduced by AVAS treatment. **a** Representative images of immunohistochemistry for Aβ deposition (MOAB-2) (top) and amyloid staining (Thio-S) (bottom) for VC (left) and AVAS (right) treatment. Scale bars: 1000 μm. **b** Quantification of % area of Aβ deposition in the CX. **c** Quantification of % area of amyloid deposition in the CX. **d** Aβ42 extraction profile from the CX measured by Aβ42 ELISA. **e** Soluble oligomeric Aβ (oAβ) from the soluble extraction fraction measured by oAβ ELISA. Data are expressed as mean ± SEM (*n* = 6–10), analyzed by Student’s *t* test, *p* < 0.05, ✝ = vs. VC

AVAS treatment reduced both Aβ and amyloid deposition. Figure 5a shows representative 10X sagittal images from VC (left) and AVAS (right) treated mice immunostained with the anti-Aβ antibody MOAB-2 (top) to detect Aβ deposition and stained with Thio-S (bottom) to detect amyloid. Both Aβ (Fig. 5b) and amyloid deposition (Fig. 5c) are significantly reduced by ~ 50% in the CX of AVAS vs. VC-treated mice.

By Aβ42 ELISA, Aβ42 in the soluble TBS, detergent TBSX, and insoluble FA extraction fractions are significantly reduced in AVAS vs. VC (Fig. 5d). The decrease in the insoluble FA extraction fraction, primarily Aβ42 from the insoluble amyloid plaques, is consistent with the decrease in Aβ deposition (Fig. 5a). Importantly, AVAS reduced soluble oAβ levels by 50% compared to VC (Fig. 5e), consistent with the 50% decrease in soluble Aβ42 in the TBS extraction fraction (Fig. 5d).

### Neuroinflammation Is Reduced by AVAS Treatment

**Fig. 6:**
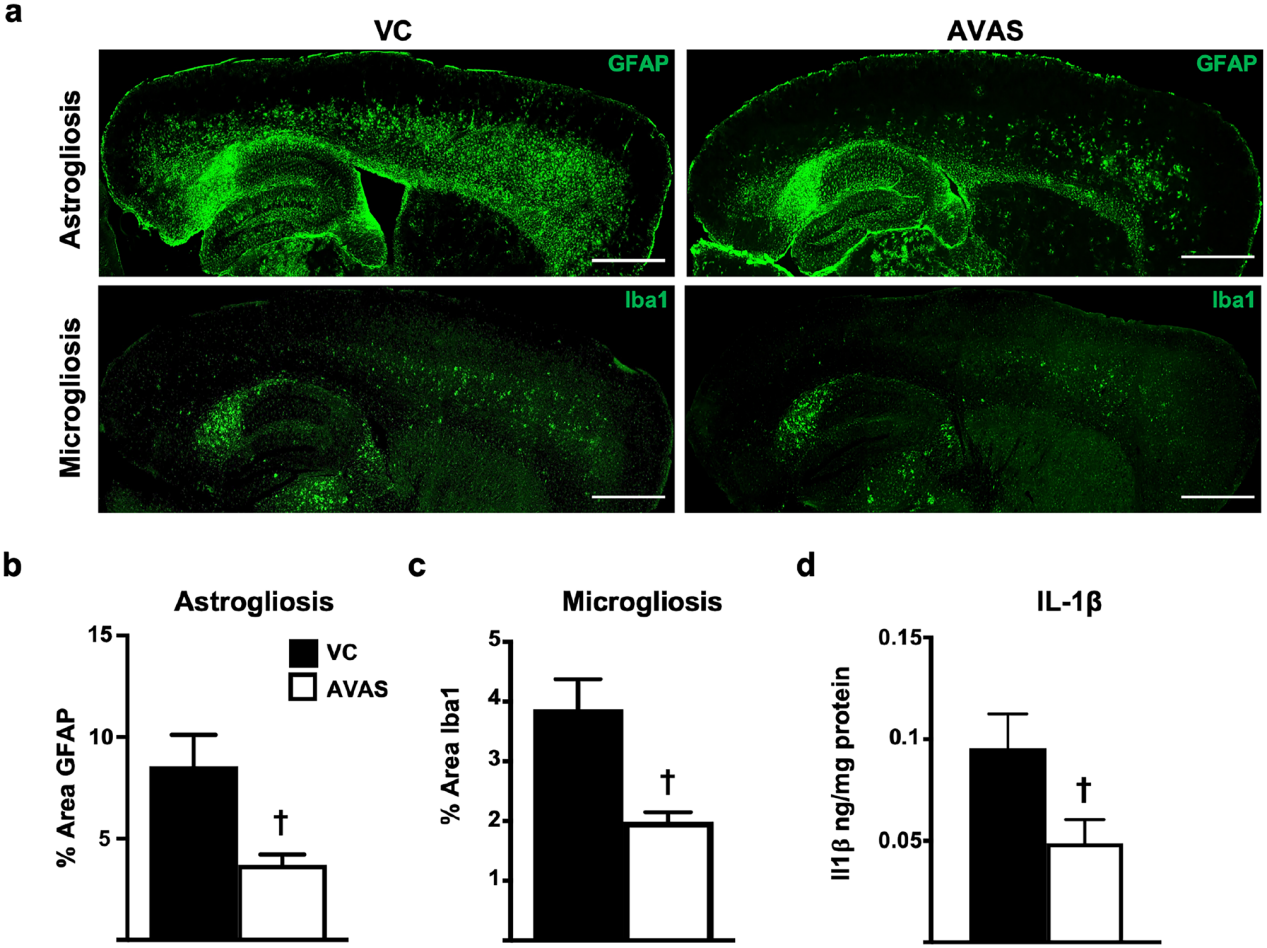
Neuroinflammation is reduced by AVAS treatment. **a** Representative images of immunohistochemistry for astrogliosis (GFAP) (top) and microgliosis (Iba1) (bottom) for VC (left) and AVAS (right) treatment. Scale bars: 1000 μm. **b** Quantification of % area of astrogliosis in the CX. **c** Quantification of % area of microgliosis in the CX. **d** by IL-1β ELISA. Data are expressed as mean ± SEM (*n* = 6–10), analyzed by Student’s *t* test, *p* < 0.05, ✝ = vs. VC

To investigate the effect of ACAT inhibition on neuroinflammation, astrogliosis and microgliosis were analyzed in male E4FAD mice after AVAS treatment. Figure 6a shows representative 10X sagittal images of from VC (left) and AVAS (right) treated mice immunostained with GFAP (top) to detect astrogliosis and Iba1 (bottom) to detect microgliosis. Both astrogliosis (Fig. 6b) and microgliosis (Fig. 6c) are significantly reduced by 50% in the CX in AVAS vs. VC treated mice. In addition, an ELISA for IL-1β, a key proinflammatory cytokine secreted by both astrocytes and microglia [104], revealed a 50% decrease in the CX with AVAS vs. VC-treated mice (Fig. 6d). Thus, AVAS exhibited both target engagement and efficacy, as well as inhibition of Aβ deposition and solubility, and neuroinflammation.

### ApoE4 Levels, Lipidated apoE, and ABCA1 Levels Are Not Affected by AVAS Treatment

**Fig. 7:**
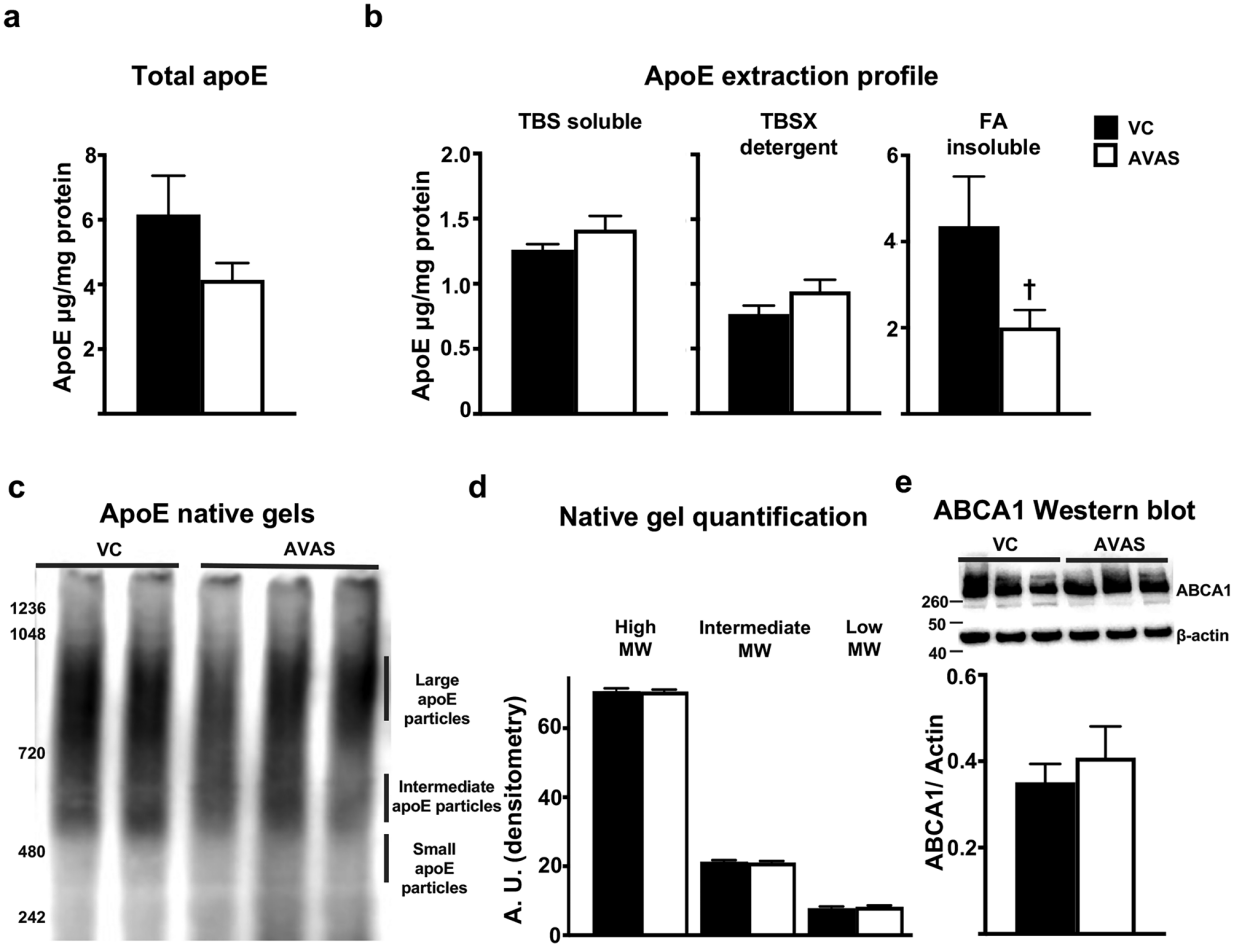
ApoE4 levels, lipidated apoE4, and ABCA1 levels are not affected by AVAS treatment. **a** Total apoE and **b** apoE extraction profile from the CX measured by apoE ELISA. **c** Native gel for apoE. **d** Quantification of apoE particles by size from native gel. **e** Western blot (top) for ABCA1 levels in the CX, normalized to β-actin for quantification (bottom). Data are expressed as mean ± SEM (*n* = 6–10), analyzed by Student’s *t* test, *p* < 0.05, † = vs. VC

AVAS treatment does not have a significant effect on total apoE4 levels (Fig. 7a). Importantly, there is no difference in the levels of apoE4 extracted in the TBSX fraction from AVAS vs. VC-treated mice (Fig. 7b). The protocol for this sequential protein extraction method was originally optimized to extract lipoprotein-associated apoE to the TBSX fraction by using 1%Triton X-100, a non-ionic detergent that is less stringent compared to SDS or Tween 20, allowing the intact lipoproteins to extract in this fraction [99]. Thus, the lack of an AVAS-induced increase in apoE4 levels in the TBSX extraction fraction is evidence that apoE4 lipidation did not increase. To further interrogate whether AVAS effects the lipidation of apoE4-lipoproteins, apoE native gels were run (Fig. 7c) and no changes in the high, intermediate, or low molecular weight particles were observed with AVAS vs. VC-treated mice (Fig. 7d). In the insoluble-FA fraction, there is a 50% reduction in apoE4 with AVAS treatment, consistent with the AVAS-induced reduction in Aβ deposition (Fig. 5a–c) and insoluble-FA Aβ42 (Fig. 5d), as apoE is known to seed, or co-deposit, with Aβ in plaques. As measured by Western blot and normalized to β-actin (Fig. 7e), ABCA1 levels did not change with AVAS treatment, evidence that cholesterol transport to the apoE4-lipoproteins was not increased [52]. These data are evidence that our hypothesis for the mechanism by which AVAS effects AD pathology likely does not involve lipidation of apoE4-lipoproteins.

### APP Processing Is Reduced by AVAS Treatment

**Fig. 8:**
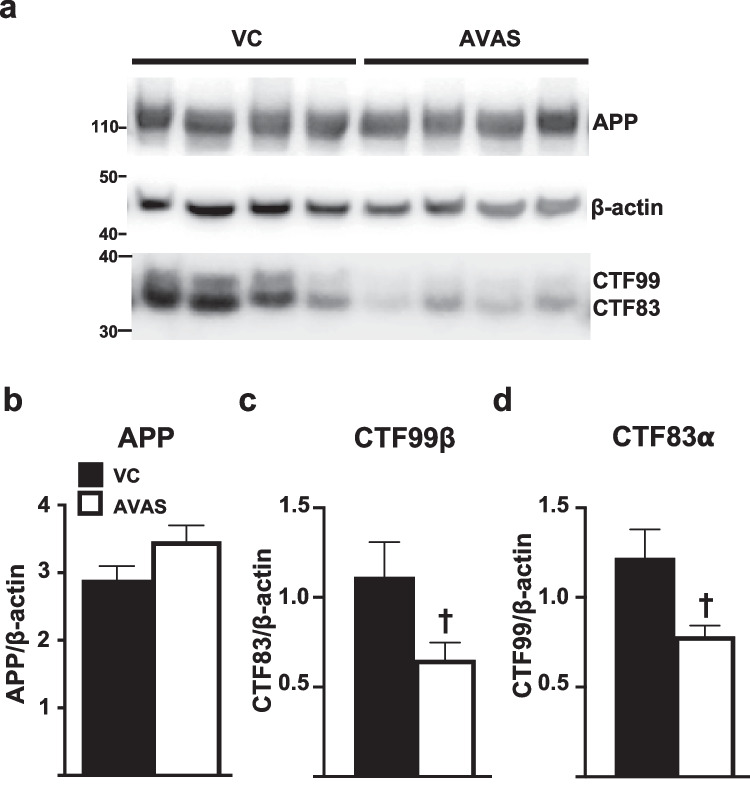
APP processing is reduced by AVAS treatment. **a** Western blot of APP processing to APP-CTFs. APP, CTFα, and CTFβ normalized to β-actin for quantification; **b** APP, **c** CTFα, and **d** CTFβ. Data are expressed as mean ± SEM (*n* = 4), analyzed by Student’s *t* test, *p* < 0.05, ✝ = vs. VC

We next tested the hypothesis that AVAS-induced inhibition of AD pathology is mediated by a decrease in APP processing by β-amyloid cleavage enzyme (β-secretase), reducing Aβ levels. To evaluate if this pathway is modulated by AVAS in E4FAD mice, TBSX brain fractions were analyzed by Western blot for full length APP and APP-CTF (Fig. 8a). APP, CTFα, and CTFβ normalized to β-actin for quantification. APP levels do not change with AVAS treatment (Fig. 8b). AVAS induced a 50% decrease in both APP-CTF83 (Fig. 8c, α-secretase/non-amyloidogenic cleavage) and APP-CTF99 (Fig. 8d, β-secretase/amyloidogenic cleavage) compared to VC-treated mice. Thus, AVAS treatment reduces APP processing, reducing the production of Aβ, consistent with previous studies using FAD-Tg mouse models expressing m-apoE [76, 105].

## Discussion

In pre-clinical studies, regulation of brain cholesterol levels through different strategies has successfully reduced Aβ pathology. Examples of these strategies include (1) reducing *de novo* synthesis of cholesterol by inhibiting the rate limiting enzyme HMG-CoA reductase [106], (2) increasing cholesterol turnover via the activation of the rate limiting enzyme CYP46A1 [107–109], and (3) enhancing cholesterol efflux via increasing levels and activity of ABCA1 [19, 46, 53, 110]. Interestingly, interventions that promote these processes reduce Aβ pathology, including increasing ER-FC via inhibiting CE formation by ACAT1 inhibition [74, 75, 103, 111].

For an AD therapeutic, efficacy is defined as enhanced or retained cognition. In this study, surrogate efficacy was defined by MWM performance in acquisition (learning) and probe (memory) trials and levels of post synaptic proteins. With MWM, previous studies demonstrate that ACAT inhibition improves MWM learning [75]. While we did not see an overall improvement during MWM acquisition trials with AVAS treatment, the latency to platform on day 1 was lower in AVAS- vs. VC-treated mice, suggesting that the AVAS treatment enhanced learning on that day only; the latency to platform was not significantly different between VC- and AVAS-treated mice for days 2–5 (Fig. 3b), an effect observed with other AVAS-treated FAD-Tg models [75]. However, with the probe trial for memory (platform removed), the latency to the platform and target quadrant was significantly less for AVAS-treated compared to VC-treated mice (Fig. 3c), a phenomenon previously reported in the literature for FAD-Tg mouse models [112–117]. An improvement in memory is consistent with the effect of memory measured by CFC in APP/ACAT^−/−^ mice [73]. Importantly, we demonstrate AVAS-induced increases in the levels of postsynaptic proteins PSD95 and drebrin (Fig. 3d).

In the current study, although AVAS treatment did not modify the cholesterol-specific plasma lipoprotein profile (Fig. 2a), brain-specific readouts did change. AVAS induced a significant reduction in LD, indirectly demonstrating target engagement (Fig. 4). LD are composed of neutral lipids, primarily CE and TAG, thus our data are consistent with previous findings that inhibition of ACAT reduces brain CE levels [75, 76]. These data suggest that the AVAS-specific effects observed in this study are confined to the CNS, independent of potential peripheral effects.

To evaluate if the observed surrogate efficacy and indirect target engagement are related to a reduction in AD pathology, we assessed Aβ pathology and neuroinflammation after AVAS treatment in E4FAD mice. AD pathology is reduced by ACAT inhibition, consistent with previous studies. Specifically, we show a reduction in soluble oAβ, soluble and insoluble Aβ42 levels, Aβ deposition, amyloid deposition, and neuroinflammation (Figs. 5 and 6) [75, 76]. Remarkably, this occurs in a FAD-Tg-mouse expressing h-apoE4, not m-apoE, and without modifying the lipidation state of apoE-particles (Fig. 7).

AVAS-induced inhibition of peripheral cholesterol esterification was originally a therapeutic target for atherosclerosis as it facilitated cholesterol efflux to apoA1-HDL in plasma, thus reducing the development of foam cells from macrophage uptake of oxidized LDL. This decrease in endothelial cell disruption and atherosclerotic plaque development was dependent on apoA1 as the plasma cholesterol acceptor [57–63]. However, little is known about the effect of ACAT inhibition of cholesterol efflux in the brain where apoE will be the primary cholesterol acceptor. To investigate this effect in vitro and in vivo, it is important to consider the difference between h-apoE and m-apoE. While humans express three primary isoforms that vary at only two of the 299 amino acids, mice express one form of apoE, the same as apoE4 at residues Arg^112^/Arg^158^, although the homology between m- and h-apoE is only 70% [118]. M-apoE and h-apoE differ in both structure [119] and functions ranging from lipid binding and transport, to effects on AD pathology [22, 84, 85, 119, 120].

Driven by the hypothesis that ACAT inhibition by AVAS will increase ABCA1-mediated FC efflux to apoE-particles in the brain, we examined the effect of ACAT inhibition in vitro on *APOE4* glia cultures. This study demonstrated an AVAS-induced increase in apoE4 levels in the media compared to VC (Fig. 1b), likely by an increase in apoE4 stability from increased lipidation of the apoE4-particles. Conversely, AVAS treatment in E4FAD mice does not appear to modify brain apoE levels or lipidation in either the plasma (Fig. 2) or the brain (Fig. 7). This may be the result of additional regulatory pathways acting in vivo and not in vitro as shown previously with ACAT inhibition exhibiting a cell-specific response, either inhibition or induction of cholesterol efflux and ABCA1 levels [57–61, 103, 121–124]. In vitro, ACAT inhibition increases ABCA1 levels and cholesterol efflux of cholesterol-loaded macrophages, while decreasing ABCA1 levels in adipocytes [125] and decreasing ABCA1 levels, lipid catabolism, and cholesterol efflux in *APOE4* microglia [126–128], thus decreasing the capacity to lipidate apoE4-particles [129]. Potentially important for the EFAD model, *APOE4* astrocytes chronically exposed to fatty acids (a possible mimic for aged astrocytes rich in LD) produce TAG-rich apoE-particles rather than cholesterol-rich particles [130]. It is possible that the drastic reduction in LD after AVAS treatment (Fig. 4) is the result of LD-CE loss, in the absence of sufficient levels of LD-TAG for formation of apoE4-TAG particles.

The primary hypothesis for the AVAS-induced reduction in AD pathology is a decrease in APP processing. Kovacs et al. described this mechanism, centered on a novel pathway in which APP is palmitoylated at the ER for mobilization to the PM lipid rafts to be processed by γ and β secretases [41]. While the specific mechanism remains unclear, ACAT inhibition significantly reduces palmitoylated APP in lipid rafts, thus reducing APP processing [75–77, 105, 131]. Although we did not measure APP palmitoylation or APP localization to lipid rafts, we did see a reduction in both amyloidogenic and non-amyloidogenic processing of APP, with no change in total APP (Fig. 8) [75, 76]. Thus, the reduction of AD pathology in EFAD mice after AVAS treatment is predominantly due to a reduction in APP processing/Aβ secretion.

Another mechanism for the AVAS-induced reduction in Aβ pathology is microglia-driven phagocytosis. Chang et al. propose that, in addition to reducing Aβ production, cholesterol mobilization by ACAT inhibition in microglia can promote autophagy and lysosomal biogenesis, leading to an increased clearance of oAβ [69–71, 77]. However, Aβ degradation by autophagosome formation has been reported for the ACAT potent inhibitor K604 and in ACAT-KO [71], suggesting that autophagocytic activity is present predominantly with the total inhibition of ACAT activity. Further, microglia from AD patients and *APOE4* carriers exhibit impaired autophagy [132–134]; thus, the cholesterol mobilization by ACAT-inhibition may not be enough to overcome an already impaired process.

This study confirms the central role of lipid homeostasis in modifying AD pathology. Overall, our results suggest that the AVAS-induced redistribution of cholesterol within in the cell is sufficient to modify LDs and lipid raft formation/distribution, resulting in increased synaptic function and reduced APP processing. However, these AVAS-induced changes are not sufficient to increase lipid efflux and modify apoE4-particles. Further, evidence suggests that the LD are rich in CE and poor in TAG, thus not supporting the formation of apoE4-TAG particles [130]. Although AVAS failed phase III clinical trials for cardiovascular efficacy [101], it has an acceptable safety profile [135, 136] and is currently being proposed as an anti-cancer agent [137]. The efficacy and potential for disease modification observed in this study with human apoE4 supports further testing of the ACAT-inhibitor AVAS for treatment of AD. In the future, it will be important to evaluate AVAS efficacy in the context of the universal biological variables (UBV) of AD risk, specifically age, *APOE*, and sex, thus understanding the effect of AVAS in the presence of apoE3 vs. apoE4, using both male and female and young vs. old EFAD mice. For instance, we cannot eliminate the possibility that AVAS treatment may increase apoE3 lipidation and further decrease Aβ levels via a clearance pathway within an *APOE3* context [138].

## Supplementary Information

Below is the link to the electronic supplementary material.

Supplementary file1 (PDF 31 kb)

Supplementary file2 (PDF 561 kb)

Supplementary file3 (PDF 597 kb)

Supplementary file4 (PDF 544 kb)

Supplementary file5 (PDF 552 kb)

Supplementary file6 (PDF 534 kb)

Supplementary file7 (PDF 588 kb)

Supplementary file8 (PDF 525 kb)

Supplementary file9 (PDF 562 kb)

Supplementary file10 (PDF 579 kb)

Supplementary file11 (PDF 612 kb)

## Data Availability

The data that support the findings of this study are available from the corresponding author, GRJT, upon reasonable request.
