# Supplementary material for: Inhibition of ACAT as a Therapeutic Target for Alzheimer’s Disease Is Independent of ApoE4 Lipidation
Source: Neurotherapeutics. 2023 May 8;20(4):1120–37. doi: 10.1007/s13311-023-01375-3 (PMC10457278; doi:10.1007/s13311-023-01375-3)
Supplement: Supplementary file 1 — Supplementary file1 (PDF 31 kb) [file 13311_2023_1375_MOESM1_ESM.pdf]

Table S1. Reagent specifications

Reagent	Vendor/catalog #	Concentration
AVAS	APExBIO Tech LLC (A4318)	-
Triton X-100	Sigma T8532	-
Bradford Reagent	Fisher (20830002-2)	-
BCA Protein assay kit	ThermoFisher (23227)	-
**Tissue culture**		
MTT Reagent A	Millipore (CT01-5)	5%
**Histochemistry**		
LipidSpot 610	Biotium (70069-T)	1X
Thio-S	Sigma (T1892)	0.5%
**α**-Aβ: MOAB-2	In-house antibody (stock at at 1mg/mL)	1:500
GFAP	Dako (Z0334)	1:500
Iba1	Wako (019-19741)	1:1000
**Native gel**		
4–20% Tris-glycine gels	ThermoFisher (WXP42026BOX)	-
Immobilon™-P PVDF Membrane	ThermoFisher (IPVH00010)	-
Ponceau S	VWR (76347-938)	-
NativeMark™ Unstained Protein Standard	ThermoFisher (LC0725)	-
Pierce™ ECL Western Blotting Substrate	ThermoFisher (32106)	-
**Western blot**		
NuPAGE™ 4 to 12%, Bis-Tris gels	ThermoFisher (WG1403BOX)	-
PVDF membrane/ filter paper	ThermoFisher (LC2002)	-
**α**-PSD95	Abcam (ab18258)	1:1000
**α**-ABCA1	Novus (NB400-105)	1:500
**α**-Drebrin	Abcam (NC1506134)	1:1000
**α**-β-actin	Sigma (A2228)	1:5000
**α**-β-tubulin	ThermoFisher (PA1-41331)	1:5000
**α**-fl-APP (22C11)	ThermoFisher (14-9749-82)	1:500
**α**-C-APP (A8717)	Sigma (A8717)	1:1000
**ELISA**		
oAβ in-house: **α**-Aβ: MOAB-2 (coating)	In-house antibody (stock at at 1mg/mL)	1:200
oAβ in-house: Biotinylated **α**-Aβ: MOAB-2 (detection)	In-house antibody (stock at at 1mg/mL)	1:5000
apoE in-house: **α**-apoE (coating)	Millipore (AB947)	1:2000
apoE in-house: **α**-apoE (detection)	Meridian (K74180B)	1:5000
apoE in-house: apoE (STD)	Creative BioMart Inc (ApoE3-3562H)	200 ng/mL
Human Aβ42 kit	ThermoFisher (KHB3442)	-
Mouse IL-1β kit	Invitrogen (KMC0012)	-
